# Clinical characteristics in schizophrenia patients with or without suicide attempts and non-suicidal self-harm - a cross-sectional study

**DOI:** 10.1186/1471-244X-13-255

**Published:** 2013-10-09

**Authors:** Erlend Mork, Fredrik A Walby, Jill M Harkavy-Friedman, Elizabeth A Barrett, Nils E Steen, Steinar Lorentzen, Ole A Andreassen, Ingrid Melle, Lars Mehlum

**Affiliations:** 1 National Centre for Suicide Research and Prevention, Institute of Clinical Medicine, University of Oslo, Sognsvannsveien 21, 0372 Oslo, Norway; 2 Division of Mental Health and Addiction, Oslo University Hospital, 0424 Oslo, Norway; 3 American Foundation for Suicide Prevention, New York, USA; 4 Institute of Clinical Medicine, University of Oslo, 0318 Oslo, Norway; 5 Department of Psychiatry, Diakonhjemmet Hospital, 0319 Oslo, Norway

**Keywords:** Suicide attempt, Non-suicidal self-harm, Self-harm, Schizophrenia, Schizoaffective, Risk factor, Depression

## Abstract

**Background:**

To investigate whether schizophrenia patients with both suicide attempts and non-suicidal self-harm have earlier age of onset of psychotic and depressive symptoms and higher levels of clinical symptoms compared to patients with only suicide attempts or without suicide attempt.

**Methods:**

Using a cross-sectional design, 251 patients (18–61 years old, 58% men) with schizophrenia treated at hospitals in Oslo and Innlandet Hospital Trust, Norway, were assessed with a comprehensive clinical research protocol and divided into three groups based on their history of suicide attempts and non-suicidal self-harm.

**Results:**

Suicide attempts were present in 88 patients (35%); 52 had suicide attempts only (29%) and 36 had both suicide attempts and non-suicidal self-harm (14%). When compared with nonattempters and those with suicide attempts without non-suicidal self-harm, patients with both suicide attempts and non-suicidal self-harm were more frequently women, younger at the onset of psychotic symptoms, had longer duration of untreated psychosis, and had higher levels of current impulsivity/aggression and depression. Patients with both suicide attempts and non-suicidal self-harm were more likely to repeat suicide attempts than patients with suicide attempts only.

**Conclusions:**

Patients with both suicide attempts and non-suicidal self-harm had different illness history and clinical characteristics compared to patients with only suicide attempts or patients without suicidal behavior. Our study suggests that patients with both suicide attempts and non-suicidal self-harm represent a distinct subgroup among patients with schizophrenia and suicidal behavior with their early onset of psychotic symptoms, high rate of repeated suicidal behavior and significant treatment delay.

## Background

Suicide is a significant problem in schizophrenia and suicide attempts (SA) are common [1-3]. Some risk factors for SA are shared with other populations, including depression, hopelessness, substance misuse and suicidal ideation [4]. Other risk factors for SA are more specific to this patient group and include more relapses, a long duration of untreated psychosis (DUP), hallucinations, and an earlier age of onset, although for the latter the association has been inconsistent across studies [4-8]. Furthermore, distressing psychotic symptoms might trigger SA, especially among those already at risk for suicidal behavior [9,10]. Suicidal behavior has been studied extensively in schizophrenia, but we still lack knowledge about the pathways that may serve as signs of persons at particular risk within this high risk group.

In particular, we have little knowledge about the impact of non-suicidal self-harm (NSSH) on suicidal behavior in this group. The limited research has focused on extreme self-injury such as self-enucleation, which is extremely rare [11]. More common forms of NSSH, such as repeated cutting, burning and self-hitting are frequently reported in other diagnostic groups with high risk for completed suicide, but have not received notable attention in studies of patients with schizophrenia [12]. Importantly, NSSH has been found to be an independent risk factor for both attempted [13] and completed suicide [14] in other clinical populations. Both self-reports, laboratory studies and clinical observations indicate that regulation of negative affect is an important mechanism underlying NSSH [15]. Studies from non-psychotic clinical samples also indicate that individuals with a history of both SA and NSSH display more severe symptoms than individuals with SA only [16-18]. Stanley and colleagues [17] found that patients with cluster B personality disorders who had a history of both SA and NSSH tended to be more depressed, have more persistent suicidal ideation, and more symptoms of affective instability and impulsivity than individuals with SA only.

Dividing patients into risk groups according to behavior is one way to increase our understanding [19]. Based on this, NSSH may be an important pathway to suicidal behavior also in schizophrenia patients. One explanation for the inconsistent association between age of onset and suicide attempts in schizophrenia may be that onset of clinical symptoms (psychotic and depressive) in adolescence will increase the risk of suicide attempts only in the subgroup of patients that are already more prone to use self-harm to regulate emotions. In a previous study we have shown that the presence of self-harm (encompassing both SA and NSSH) is associated with younger age of onset of schizophrenia [20]. Self-harm was also associated with depression, current suicidal ideation in both genders, and impulsive aggression in women. This study protocol did not initially differentiate between SA and NSSH. In the current study, however, we have used an expanded protocol assessing life-time SA and NSSH separately. Based on the hypothesis that patients with both SA and NSSH represent a separate subgroup characterized by earlier onset and more affective and impulsive symptoms, the aim of the present study was to investigate: 1) Whether patients with SA + NSSH have earlier onsets of clinical symptoms (psychotic and/or depressive) and 2) Whether patients with SA + NSSH report more impulsive aggression, suicidal ideation and depressive symptoms than SA patients without NSSH or patients without SA.

## Methods

The study was part of the Thematically Organized Psychosis (TOP) Study including patients with psychotic disorders from in- and outpatient clinics at the major hospitals in Oslo and Innlandet Hospital Trust, Norway. Recruitment of participants, inclusion criteria and clinical assessments are described briefly below. More detailed information can be found elsewhere [6,20]. The study was approved by The Regional Committee for Medical and Health Research Ethics South East, Norway and by the Norwegian Data Protection Agency.

### Participants

The current study sample consisted of 251 patients, 139 outpatients (55%) and 112 inpatients, with a DSM-IV narrow schizophrenia spectrum disorder (DSM IV 295.xx) consecutively included between April 2007 and November 2010. The age range of the participants was 18–61 years, with a mean age of 30.1 years (SD = 9.8 years). Fifty-eight per cent of subjects were men (*n =* 145), the majority of participants (82%) were of European origin and 81% were single.

### Clinical assessments

Clinical psychologists or medical doctors, with formal training in use of the assessment protocol, interviewed the patients when in a stable phase. The Structured Clinical Interview for DSM-IV Axis I disorders (SCID-I) [21] was used for diagnostic purposes. Mean overall kappa for SCID diagnoses as assessed in the training course was 0.77. Age of onset was defined as age at the first SCID-verified psychotic episode. Clinical symptoms were assessed using the Positive and Negative Syndrome Scale, PANSS [22]. All interviewers participated in inter-rater reliability testing that entailed rating of patient videos. Inter-rater reliability was acceptable with intra-class correlation coefficients [23] for PANSS subscales ranging from 0.71 to 0.73. The PANSS item G14 (Disordered regulation and control of action on inner urges/emotions) was used as a proxy for impulsive aggression in the past week. Suicidal ideation last week was measured using five items from the InterSePT scale for suicidal thinking [24]. Duration of untreated psychosis was measured as time from psychosis onset until start of adequate treatment for psychotic disorder [25].

**Figure 1:**
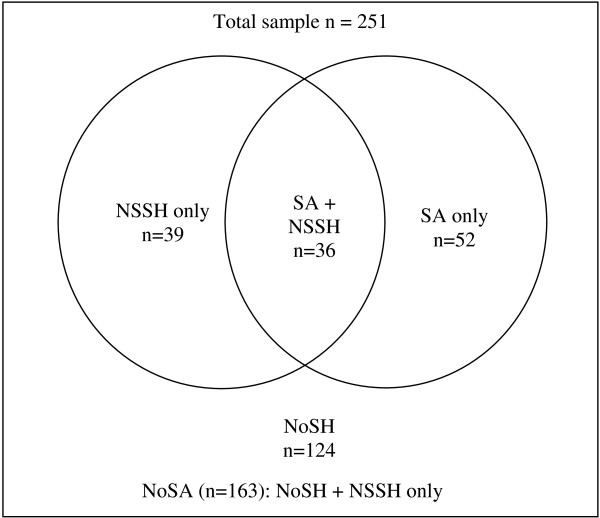
**Grouping of patients according to life-time prevalence of self-harm behaviors.** NSSH: non-suicidal self-harm (n = 75) SA: suicide attempt (n = 88). NoSH : no self-harm (n = 124) NoSA : no suicide attempt (n = 163).

The concept self-harm (SH) [26] was subdivided into SA, defined as ‘self-harm with the intent to die’, and NSSH, defined as ‘self-harm without suicide intent’. NSSH thus encompasses both non-suicidal self-injury and self-poisoning for purposes of self-injury without suicidal intent. Information on lifetime episodes of self-harm was based on a semistructured interview including the following question adopted from a previous European study of self-harm (CASE-study): “Have you ever deliberately taken an overdose (e.g., of pills or other medication) or tried to harm yourself in some other way (such as cut yourself)?” [26,27]. The response options were “no”, “yes” and if yes, “the specific number of times” with the follow-up question: “how many times did you try to kill yourself?” (SA) and “How many times did you harm yourself without a wish to die” (NSSH). The presence of any suicide intent led to classification of the behavior as a suicide attempt, also in the presence of seemingly contradictory additional reasons given for the behavior (such as “I wished to get help from someone”). In addition to the self-harm question, the classification was based on an open description of the participants’ most recent episode of self-harm, whether they considered the behavior a suicide attempt (yes, no, uncertain), and on answers to questions about what they wanted to achieve with the act (“I wished to die” (yes/no), “I wished to get help from someone” (yes/no), “I wished to escape an unbearable emotion” (yes/no), ”Other reasons (describe)”). The principal author reviewed all classifications of self-harm variables and in the few cases (n = 6) with a discrepancy, a consensus was reached with the senior author. This defined three groups relative to the presence of SA: 1) NoSA (patients with no SH and NSSH only), 2) SA only (participants with history of SA, but no NSSH) and 3) SA + NSSH (participants with both SA and NSSH) (see Figure 1).

### Statistical analyses

Group differences were analyzed using Chi-square tests for categorical variables, one-way ANOVAs (post-hoc Scheffé’s tests) for normally distributed continuous variables and Kruskal-Wallis test (post hoc Mann–Whitney U test) for non-normally distributed continuous variables. Pairwise comparisons were performed comparing the SA + NSSH group with the SA only and with the NoSA groups, based on the research questions. All tests were two-tailed with α < 0.05. Two multinomial logistic regression models were generated to answer the two research questions. Backward stepwise procedures (p in = 0.05: p out = 0.10) were used to search for the models that best differentiated between participants with SA + NSSH, SA only, and NoSA history. DUP and impulsive aggression (G14) were skewed and dichotomized at the median (DUP > 52 weeks/G14 > 1) in the regression analysis. We also included variables that were differentially distributed over groups and thus could confound relationships. Current age and age of first psychotic symptoms were highly correlated and since age of first psychotic symptoms was of primary interest, this measure was retained in the models. We used the PASW statistics v.18 software (SPSS Inc., Chicago, Illinois).

## Results

### Rates and characteristics of NSSH and SA

A total of seventy-five patients (30%) reported one or more episodes of NSSH. The median number of NSSH episodes was six (min = 1, max ≥ 50), 22 patients (9% of total sample) reported 50 episodes or more. Women were significantly more likely to report at least one NSSH episode (43% (n = 46) vs. 20% (n = 29), χ2 = 16.00, DF = 1, p < 0.001). When the most recent self-harm episode was NSSH (n = 54), it had little or no risk of death (n = 52/96%), the behavior had been planned for less than an hour in 35 cases (65%), and the most commonly used method was cutting (n = 42/78%). Among those with SA + NSSH, almost all patients reported cutting as method, when the most recent episode was NSSH (n = 17/94%), indicating that NSSH in this group primarily consists of self-injury. One or more episodes of SA were present in 88 patients (35%), the median number of SA among these was two (min = 1, max = 7). Significantly more women reported history of SA (47% (n = 50) vs. 26% (n = 38), χ2 = 11.82, DF = 1, p = 0.001). When the most recent self-harm was SA (n = 63), the lethality was rated as moderate to high in 45 cases (77%), and the most frequent method was self-poisoning (n = 34/54%). Patients with a lifetime history of NSSH were significantly more likely to report SA (n = 36/48%) than patients without NSSH (n = 52/30%, χ2 = 7.9, DF = 1, p = 0.005). Fifty-two patients (21%) had a history of SA only while 36 (14% of total) had a history of SA + NSSH. The SA + NSSH group was significantly more likely to have repeated SA (n = 28/78%) compared to the SA only group (n = 17/33%, χ2 = 17.3, DF = 1, p < 0.001). Patients with SA + NSSH were significantly more often female and younger at the time of participation than patients with SA only or NoSA (Table 1).

**Table 1:** Sociodemographic variables and illness history according to history of self-harm behaviour

	**Overall n = 251**^**a**^	**Suicide attempt n = 88**		**No suicide attempt**	**Statistics (DF)**	**Post hoc**^**e**^
		**Group 1**	**Group 2**	**Group 3**		
		**SA + NSSH**	**SA only**	**NoSA**		
		**n = 36**	**n = 52**	**n = 163**		
Age (years), mean (SD)	30.1 (9.8)	24.5 (6.3)	33.5 (10.3)	30.3 (9.9)	*F =* 9.5 (2, 250)***^b^	1 < 2, 3
Women, n (%)	106 (42)	26 (72)	24 (46)	56 (34)	*χ*^*2*^ = 17.7 (2)***^c^	1 ≠ 2, 3
Age of first psychotic symptoms, mean (SD)	22.8 (7.7)	17.6 (6.4)	24.3 ( 9.2)	23.5 (7.0)	*F* = 10.7 (2, 249)***^b^	1 < 2, 3
Age of first treatment for psychiatric disorder, mean (SD)	24.6 (9.0)	19.9 (5.6)	25.5 (9.7)	25.3 (9.2)	*F* = 6.0 (2, 244)**^b^	1 < 2, 3
Age of first treatment for psychoses, mean (SD)	25.6 (7.6)	22.5 (5.0)	28.0 (8.9)	25.5 (7.5)	*F* = 5.6 (2, 239)**^b^	1 < 2
First treatment for other reason than psychosis, n (%)	70 (29)	21 (58)	17 (33)	32 (20)	χ^2^ = 21.8 (2)***^c^	1 > 2, 3
Duration of untreated psychoses (weeks), median (min-max)	52 (0–2040)	181 (0–1352)	32 (1–2040)	28 (0–1248)	*K* = 14.3 (2)**^d^	1 > 2, 3
Depressive episode, lifetime n (%)	129 (52)	27 (75)	42 (81)	60 (37)	χ^2^ = 38.9 (2)***^c^	1 > 3
Depressive episode, first symptoms before the age of 18, n (%)	54 (22)	20 (57)	14 (29)	20 (13)	χ^2^ = 34.1 (2)***^c^	1 > 2, 3

Levels of statistical significance: ns = not significant, ** = p < 0.01, *** = p < 0.001.

SA: Suicide attempt. NSSH: Non-suicidal self-harm.

^a^Data missing for 0–11 patients. Percentages based on valid observations, ^b^F = One-way ANOVA test, ^c^χ2 = Pearson chi square test, ^d^K = Kruskal-Wallis test, ^e^Planned pairwise comparisons of Group 1 (SA + NSSH) vs Group 2 (SA only) and Group 1 (SA + NSSH) vs Group 3 (NoSA) (Scheffe’s test, Pearson chi square test, Mann–Whitney U test). Only statistically significant differences (p < .05) presented.

### Illness history and medication

Patients with SA + NSSH were significantly younger at psychosis onset than participants with SA only or NoSA (mean difference 6.7 and 4.9 years respectively), and had longer DUP (Table 1). SA + NSSH patients were also significantly younger at first contact with mental health services and more likely to have had their first mental health contact for other reasons than psychotic disorder than the other patients. Furthermore, they were significantly more likely to report to have had depressive episode(s) before the age of 18.

### Current symptoms and behaviors

The SA + NSSH group had significantly higher levels of depressive symptoms and were more likely to report current impulsive aggression or suicidal ideation compared to the SA and NoSA groups (Table 2). There were no group differences in current levels of negative symptoms or total PANSS score. There was, however, a trend towards group differences on positive symptoms (p = .08), and significant group differences were observed on the delusions and hallucinatory behavior items from the positive symptoms score, with those with SA + NSSH scoring significantly higher than both other groups on current hallucinatory behavior. One third of the SA + NSSH patients did not use any antipsychotic medication; a significantly higher fraction than among NoSA patients. Regarding recent SA, there were no significant differences between the SA + NSSH and SA only groups in lethality (moderate to high: 75% vs 77%, χ2 = 0.0, DF = 1, p = 0.854), degree of hospital treatment (41% vs 49%, χ2 = 0.3, DF = 1, p = 0.583), degree of premeditation (less than an hour: 41% vs 43%, χ2 = 0.0, DF = 1, p = 0.887), or in method used (self-poisoning: 59% vs 51%, χ2 = 0.3, DF = 1, p = 0.583).

**Table 2:** Current symptoms, medication and behavior according to history of self-harm behavior

	**Overall n = 251**^**a**^	**Suicide attempt n = 88**		**No suicide attempt**	**Statistics (DF)**	**Post hoc**^**e**^
		**Group 1**	**Group 2**	**Group 3**		
		**SA + NSSH**	**SA only**	**NoSA n = 163**		
		**n = 36**	**n = 52**			
Global Assessment of Functioning, mean (SD)	41.7 (10.3)	37.4 (7.4)	41.6 (8.9)	42.6 (11.0)	*F =* 3.9 (2, 250)**^b^	1 < 3
PANSS						
Total, mean (SD)	67.2 (17.6)	72.0 (15.3)	66.3 (17.2)	66.3 (18.1)	*F* = 1.6 (2, 247)^ns^^b^	
Positive symptoms, mean (SD)	16.3 (5.4)	17.9 (4.1)	16.6 (5.5)	15.8 (5.5)	*F* = 2.5 (2, 250)^ns^^b^	
- PANSS P1 Delusions	3.6 (1.4)	4.1 (1.2)	3.6 (1.2)	3.4 (1.5)	*F =* 3.7 (2, 249) *^b^	1 > 3
- PANSS P3 Hallucinatory behavior	3.0 (1.7)	3.9 (1.5)	2.9 (1.5)	2.8 (1.7)	*F =* 7.0 (2, 249) **^b^	1 > 2, 3
Negative symptoms, mean (SD)	16.7 (6.6)	15.8 (6.4)	15.6 (5.9)	17.2 (6.8)	*F* = 1.5 (2, 249)^ns^^b^	
CDSS (current depression) suicide item excluded^c^, mean (SD)	5.6 (4.7)	9.5 (5.0)	6.0 (4.6)	4.6 (4.2)	*F =* 18.2 (2, 241) ***^b^	1 > 2, 3
PANSS G14 (Impulsive aggression), median (min-max)	1 (1–6)	2 (1–6)	1 (1–4)	1 (1–4)	*K* = 24.1 (2)***^c^	1 > 2, 3
InterSePT 5 (Current suicidality)					χ^2^ = 30.4 (4)***^d^	
- No current suicidality (0), n (%)	157 (63)	13 (36)	27 (53)	117 (72)		
- Low suicidality (0.1-1.0), n (%)	75 (30)	14 (39)	20 (39)	41 (25)		
- Moderate to high (1.1-2.0), n (%)	18 (7)	9 (25)	4 (8)	5 (3)		1 > 2, 3^f^
Alcohol abuse or addiction last 6 months, n (%)	16 (7)	2 (6)	6 (12)	8 (5)	χ^2^ = 2.9 (2)^ns^^d^	
Substance abuse or addiction last 6 months, n (%)	23 (9)	4 (11)	5 (10)	14 (9)	χ^2^ =0.2 (2)^ns^^d^	
Current medication:					*X*^*2*^ = 10.9 (4) *^d^	
- No antipsychotic medication, n (%)	44 (18)	12 (33)	11 (21)	21 (13)		1 ≠ 3^g^
- Antipsychotic, no antidepressant, n (%)	150 (60)	16 (44)	27 (52)	107 (66)		
- Antipsychotic and antidepressant, n (%)	57 (23)	8 (22)	14 (27)	35 (22)		

Levels of statistical significance: ns = not significant * = p < 0.05, ** = p < 0.01, *** = p < 0.001.

^a^Data missing for 0–9 patients, percentages based on valid observations, ^b^*F* = One-way ANOVA test, ^c^K = Kruskal-Wallis test, ^d^χ^2^ = Pearson chi square test, ^e^Planned pairwise comparisons of Group 1 (SA + NSSH) vs Group 2 (SA only) and Group 1 (SA + NSSH) vs Group 3 (NoSA) (Scheffe’s test, Pearson chi square test, Mann–Whitney U test). Only statistically significant differences (p < .05) presented. ^f^ Comparing those scoring moderate to high with those with low/no current suicidality, ^g^ Comparing those with no antipsychotic medication to those with antipsychotic medication (with/without antidepressant medication).

### Multivariate analyses

In the first model we investigated the multivariate relationships between group memberships and illness history (Aim 1, i.e. ages at onset of psychosis and depression) together with potential confounding variables of this relationship (here DUP and gender). All four variables had a significant contribution to the model (Table 3). The SA + NSSH group could be differentiated from the NoSA group by a) being younger at the time of onset of their first psychotic symptoms, b) more often having had episodes of depression before the age of 18, c) being more often female and d) more often having a DUP of more than a year. The SA only group could only be differentiated from the NoSA group by more often having had episodes of depression before the age of 18. To test whether the SA + NSSH and SA only groups were significantly different on these measures, we performed identical multinomial analyses with the SA + NSSH group as reference category (see Additional file 1). These analyses confirmed that the SA + NSSH group differed significantly from the SA only group by a) being younger at first psychotic symptoms, b) being more often female and c) more often having had a DUP of more than a year. There were no significant differences between the SA + NSSH and SA only groups in having had episodes of depression before the age of 18.

**Table 3:** **Clinical characteristics according to type of self-harm behavior. Multinomial logistic regression analysis**^**a**^

	**Model 1: Age at onset and DUP**^**b**^		**Model 2: Model 1 + Current symptoms and behavior Reference category: NoSA**^**c**^	
	**Reference category: NoSA**			
	**SA + NSSH**	**SA only**	**SA + NSSH**	**SA only**
	**Adj. OR**	**Adj. OR**	**Adj. OR**	**Adj. OR**
	**(CI 95%)**	**(CI 95%)**	**(CI 95%)**	**(CI 95%)**
**Age of onset and DUP**				
Higher age of first psychotic symptoms	0.92 (0.86 – 0.99)*	1.02 (0.98 – 1.07)^ns^	0.92 (0.85 – 0.99)*	1.02 (0.98 – 1.07)^ns^
DUP > 52 weeks (1 year)	3.10 (1.20 – 8.04)*	1.09 (0.55 – 2.17)^ns^	-	-
Women	7.41 (2.73 – 20.12)***	1.53 (0.77 – 3.03)^ns^	4.63 (1.69 – 12.64)**	1.47 (0.73 – 2.99)^ns^
Depressive episode, first symptoms before the age of 18	4.82 (1.88 – 12.35)**	3.01 (1.31 – 6.95)*	3.36 (1.20 – 9.40)*	2.62 (1.08 – 6.32)*
**Current symptoms and behavior**				
PANSS G14 (Impulsive aggression) > 1			5.30 (2.00 – 14.04)**	0.93 (0.42 – 2.06)^ns^
CDSS (current depression) suicide item excluded			1.22 (1.10 – 1.35)***	1.09 (1.00 – 1.18)*

^a^The data in the columns presents the odds ratios for participants with SA + NSSH or SA only having the given characteristic compared to participants with NoSA (reference category). Levels of statistical significance: ns = not significant * = p < 0.05, ** = p < 0.01, *** = p < 0.001.

^b^Variables entered in Model 1: Age of first psychotic symptoms, Depressive episode, First symptoms before the age of 18, DUP > 52 weeks, Gender.

^c^Variables entered in Model 2: step 1 + Current medication, GAF F, CDSS (current depression), PANSS G14 (Impulsive aggression), InterSePT 5 (Current suicidality). Step 2 Model chi-square = 311.084, df = 10, p < .001. The model as a whole explained between 31% (Cox and Snell R^2^) and 37% (Nagelkerke R^2^) of the variance and correctly identified 71% of the cases. n = 233 in the final model.

In Model 2 we added current symptoms and behavior to the illness history variables entered in model 1 to address the second aim of this study. Levels of current impulsive aggression and depressive symptoms contributed to differentiate between the groups. The SA + NSSH group had higher scores on current impulsive aggression and depressive symptoms compared to the NoSA group. Current depressive symptoms also differentiated the SA only group from those with NoSA, while current impulsive aggression did not. Once current depressive symptoms were added, length of DUP no longer contributed significantly to the model (Model 2, Table 3). Current medication did not significantly differentiate between groups in the multivariate analyses. The supplementary analysis with SA + NSSH as reference category showed that the SA + NSSH group scored significantly higher on both current impulsive aggression and depressive symptoms compared with the SA only group (Additional file 1).

## Discussion

Our main findings are that a history of SA + NSSH in patients with schizophrenia is associated with an earlier onset of psychotic symptoms and a longer DUP compared to patients with SA only or NoSA. SA + NSSH were more common in females and patients with both SA and NSSH had higher levels of current impulsive aggression and depressive symptoms. Despite these differences, the groups could not be distinguished by characteristics of their suicide attempts, which were frequently quite dangerous.

To our knowledge, this is the first study to address the prevalence of NSSH and the clinical correlates of a history of both SA and NSSH as opposed to SA only or no suicide attempt history in patients with schizophrenia. We found that NSSH was relatively frequent (1/3 of the total sample having at least one episode) as was also SA + NSSH. In comparison, prevalence estimates of NSSH in mixed adult general psychiatric samples vary considerably with the type of data source. Chart review studies generally report low levels (6%) [28] while self-report questionnaire studies asking specifically about a wide range of NSSH behaviors (including scratching or skin picking) report higher rates (41 - 45%) [29,30].

The NSSH behaviors reported in the current study usually carried little death risk, were often highly repetitive, and in most cases involved cutting, consistent with findings from other adult general psychiatric samples [16,30,31]. SA + NSSH patients were younger than the other patients in our sample. Since the current sample is relatively young with short durations of treated illness, cohort effects and recall bias are not likely to explain the observed differences. We would thus argue that it is likely that SA + NSSH are more prevalent among patients with earlier onset of schizophrenia, in line with our original hypotheses.

We also found support for our hypothesis of more current impulsive aggression and depressive symptoms among patients with SA + NSSH, suggesting a link between NSSH or repeated self-harm behaviors and disturbances of affect regulation also in schizophrenia patients. This is in line with a previous finding of an association between affective variability and suicidal ideation in individuals with high risk for developing schizophrenia [32]. Depression/depressive symptoms, suicidal ideation, impulsivity and repeated self-harm behaviors are symptoms and behaviors found to be linked to each other and to underlying problems with affect regulation in other diagnostic groups, especially borderline personality disorder (BPD) [17]. Since we did not interview specifically for personality disorders it is possible that patients with SA + NSSH in the present sample displayed BPD traits in addition to schizophrenia. Studies of prodromal or recent onset schizophrenia patients show that they may experience a wide array of comorbid syndromes, including premorbid BPD [33,34]. However, the lower age of onset for psychotic symptoms in the SA + NSSH group might also indicate that the emergence of psychotic symptoms during adolescence can increase the risk of self-harm [35], perhaps to regulate affect. Severity of depressive symptoms and suicidal behavior are robust predictors of future suicide attempts in adult samples of schizophrenia patients [4,36] and a recent study of children and adolescents with first episode psychoses also found that depressive symptoms and high suicidality at baseline was associated with increased risk for suicide attempts in the follow-up period [37]. That study did not report on NSSH, but such findings highlights that the higher severity of depressive symptoms and higher current suicidality in patients with both SA and NSSH increase the risk of future suicide attempts.

Studies of first episode patients (including a subsample of the current) indicate that longer DUP is associated with suicidality [6,38,39]. The current study did not have an a-priori hypothesis regarding the role of DUP, but included the measure as part of a range of potential risk indicators. It is of clinical importance that the SA + NSSH group had significantly longer DUPs while at the same time more than half of the group reported that they had their first treatment contact with mental health services early, but for other reasons than their psychotic disorder. It is thus unlikely that the observed treatment delay is explained by unfamiliarity with mental health services. Rather we could speculate that early onset of depression, possibly in combination with repeated self-harm behaviors and/or other symptoms and behavior such as suicidal ideation and impulsive aggression may have delayed a thorough diagnostic assessment of their psychotic symptoms. From this point of view it is important to note that one third of the SA + NSSH patients currently did not use any antipsychotic medication; a significantly lower fraction than among NoSA patients.

### Strengths and limitations

Strengths: The strengths of the study include the relatively large and representative sample recruited from publicly funded catchment area based services serving all socio-economic classes as well as the use of comprehensive clinical assessments performed by qualified and trained clinical interviewers. Limitations include the cross-sectional design preventing inferences about causal relationships, the general problems with recall bias for retrospective data, the lack of data on the age of onset of SA + NSSH and the use of PANSS item G14 as a proxy measure for impulsive aggression. The relatively small size of the groups of interest increases the risk of Type II error. Findings must thus be interpreted with some caution.

## Conclusions

The results of the current study indicate that non-suicidal self-harm is highly prevalent in patients with schizophrenia, and that the subgroup with both suicide attempts and non-suicidal self-harm could constitute a distinctive subpopulation characterized by an early onset of psychotic symptoms and yet a significant delay of treatment for psychosis. The high frequency of suicide attempts combined with higher levels of impulsive aggression and depressive symptoms suggest that this group may be at increased risk for severe suicidal behavior in the future. Assessment of non-suicidal self-harm should be part of standard suicide risk assessment of schizophrenia patients. These results suggest that an early- and thorough diagnostic assessment of psychotic symptoms in individuals with both suicide attempts and non-suicidal self-harm is important to prevent delayed treatment and perhaps increased risk of repeated non-suicidal self-harm and severe suicide attempts.

## Abbreviations

BPD: Borderline personality disorder; DUP: Duration of untreated psychosis; NoSA: No suicide attempt; NSSH: Non-suicidal self-harm.; PANSS: Positive and negative syndrome scale; SA: Suicide attempt; SCID-I: The structured clinical Interview for DSM-IV Axis I disorders; SH: Self-harm.

## Competing interests

All authors declare that they have no competing interests.

## Authors’ contributions

EM, LM, FAW, JMH, IM, OAA and SL contributed to the study design and/or the writing of the protocol. EM, EAB and NES contributed to the data collection. EM conducted the literature searches and statistical analyses and wrote the first draft of the manuscript. All authors contributed to and have read and approved the final manuscript.

## Pre-publication history

The pre-publication history for this paper can be accessed here:

http://www.biomedcentral.com/1471-244X/13/255/prepub

## Supplementary Material

Additional file 1 **Clinical characteristics according to type of self-harm behavior.** Multinomial logistic regression analysis^a^. Reference category: SA + NSSH. Click here for file
