# Supplementary material for: Clinical characteristics in schizophrenia patients with or without suicide attempts and non-suicidal self-harm - a cross-sectional study
Source: BMC Psychiatry. 2013 Oct 9;13:255. doi: 10.1186/1471-244X-13-255 (PMC3852098; doi:10.1186/1471-244X-13-255)
Supplement: Additional file 1 — Clinical characteristics according to type of self-harm behavior. Multinomial logistic regression analysisa. Reference category: SA + NSSH. [file 1471-244X-13-255-S1.doc]

**Clinical characteristics according to type of self-harm behavior. Multinomial logistic regression analysis^a^. Reference category: SA+NSSH**

|  | **Model 1: Age at onset and DUP^b^**  **Reference category: SA+NSSH** | | **Model 2: Model 1 + Current symptoms and behavior**  **Reference category: SA+NSSH^c^** | |
| --- | --- | --- | --- | --- |
|  | **SA only** | **NoSA** | **SA only** | **NoSA** |
|  | Adj. OR  (CI 95 %) | Adj. OR  (CI 95 %) | Adj. OR  (CI 95 %) | Adj. OR  (CI 95 %) |
| **Age of onset and DUP** |  |  |  |  |
| Higher age of first psychotic symptoms | 1.10 (1.03-1.20)** | 1.09 (1.01-1.17)* | 1.12 (1.03-1.21)** | 1.09 (1.01-1.18)* |
| DUP > 52 weeks (1 year) | 0.35 (0.12 – 0.99)* | 0.32 (0.12 – 0.84)* | - | - |
| Women | 0.26 (0.09 – 0.75)* | 0.17 (0.07 –0.45)*** | 0.32 (0.11 – 0.96)* | 0.22 (0.08 – 0.59)** |
| Depressive episode, first symptoms before the age of 18 | 0.63 (0.22-1.77)^ns^ | 0.21 (0.08-0.53)** | 0.78 (0.25-2.41)^ns^ | 0.30 (0.11-0.84)* |
| **Current symptoms and behavior** |  |  |  |  |
| PANSS G14 (Impulsive aggression) > 1 |  |  | 0.18 (0.06-0.52)** | 0.19 (0.07-0.50)** |
| CDSS (current depression) suicide item excluded |  |  | 0.89 (0.80-0.99)* | 0.82 (0.74-0.91)*** |

^a^ The data in the columns presents the odds ratios for participants with SA only or NoSA of having the given characteristic compared to participants with SA+NSSH (reference category). Levels of statistical significance: ns = not significant * = p < 0.05, ** = p < 0.01, *** = p < 0.001

^b^ Variables entered in Model 1: Age of first psychotic symptoms, Depressive episode, First symptoms before the age of 18, DUP > 52 weeks, Gender.

^c^ Variables entered in Model 2: step 1 + Current medication, GAF F, CDSS (current depression), PANSS G14 (Impulsive aggression), InterSePT 5 (Current suicidality).

Step 2 Model chi-square = 311.084, df = 10, p <.001. The model as a whole explained between 31% (Cox and Snell R^2^) and 37% (Nagelkerke R^2^) of the variance and correctly identified 71% of the cases. n= 233 in the final model.
